# Translation and cross-cultural adaptation of Osteoporosis Knowledge Assessment Tool (OKAT) for Chinese populations in Australia

**DOI:** 10.1007/s11657-024-01404-4

**Published:** 2024-05-31

**Authors:** John Brianna Bronio, Lei Si, David Lim, Clarice Tang

**Affiliations:** 1 School of Health Sciences, Western Sydney University, Narellan Road & Gilchrist Drive, Campbelltown, NSW 2560 Australia; 2 Centre for Improving Palliative, Aged Care and Chronic Conditions Through Clinical Research and Translation, Faculty of Health, University of Technology Sydney, 15 Broadway, Ultimo, NSW 2007 Australia; 3 College of Sport, Health and Engineering, Victoria University, 370 Little Lonsdale Street, Melbourne , VIC 3000 Australia

**Keywords:** Chinese, Culturally and linguistically diverse groups, Osteoporosis, Osteoporosis Knowledge Assessment Tool (OKAT)

## Abstract

***Summary*:**

The increased prevalence of osteoporosis among Chinese-speaking communities in Australia deemed it necessary to have a culturally appropriate tool for assessing knowledge. This study describes the cultural adaption of the validated Osteoporosis Knowledge Assessment Tool (OKAT). The adapted tool is readable and understandable for diverse Chinese-speaking communities.

**Purpose:**

With an expected increasing prevalence of osteoporosis among Chinese-speaking communities in Australia, a cross-culturally adapted questionnaire is necessary to assess knowledge levels among the group. We aimed to cross-culturally adapt the Osteoporosis Knowledge Assessment Tool (OKAT) questionnaire for Chinese-speaking populations in Australia.

**Methods:**

Cross-cultural adaptation guidelines were employed to culturally adapt the OKAT to simplified Chinese. This involved translation, revision, retroversion, and expert discussion before finalizing the Chinese version of OKAT. The participants were recruited through convenience sampling from a cohort of Chinese-speaking populations who attended a bone health promotion program. The adapted questionnaire was piloted with Chinese-speaking communities in the Greater Western Sydney area for face and content validity. The adapted questionnaire was compared with the original version for response agreement using Cohen’s kappa goodness of fit. The face validity of the adapted tool was analysed through a binary scale rating for readability and understandability.

**Results:**

The cross-culturally adapted version of OKAT has a 71.8% total response agreement with the original version of OKAT. The cross-culturally adapted OKAT yielded higher total scores than the translated version. The cross-culturally adapted tool had a good face and content validity.

**Conclusion:**

The cross-culturally adapted version of OKAT improves the overall readability and understandability of the questionnaire among Chinese-speaking populations in Australia.

## Introduction

Osteoporosis is a common chronic systemic skeletal disease characterized by low bone mass density (a *T-*score of − 2.5 standard deviation, or an age, gender, body size-adjusted *Z*-score of − 2.0 or lower), deterioration of bone tissue, increased bone fragility, and susceptibility to fractures [1]. Osteoporosis poses a major public health burden in Australia due to its “silent nature” as the disease progression is often asymptomatic, underdiagnosed, and untreated until a recent or minor fracture has occurred [2, 3]. With the rapidly increasing aged population in Australia, it is reported that the burden of disease has increased to 31% in 2022, with an estimated 66% or a total of 4.74 million aged 50 and over being diagnosed with osteopenia and osteoporosis; a higher prevalence among women of Caucasian and Asian backgrounds [4–7]. While Australians with Chinese heritage currently comprised 2.3% (595,630) of Australia’s total population as of 2021 [4–7], the burden of osteoporosis among the Chinese-speaking population in Australia is expected to increase due to the continuous increase in Chinese net migration in Australia and as the population ages [4, 8–10].

Osteoporosis can be effectively managed using non-pharmacological and pharmacological treatments [11, 12]. The challenge with adherence to osteoporosis management lies in changing an individual’s behavior and beliefs towards the disease [11, 12]. In accordance with the Capability Opportunity Motivation-Behavior (COM-B) framework [13], it is known that the level of disease-specific knowledge that an individual has plays a role in impacting their capability to manage the disease. Various tools can be utilized to assess osteoporosis-related knowledge. One of them is the Osteoporosis Knowledge Assessment Tool (OKAT) developed in Australia. OKAT is a reliable and valid questionnaire comprising 20-item statements with good psychometric properties [12]. It has four basic domains, including (1) understanding symptoms and risk of fracture, (2) knowledge of risk factors, (3) knowledge of preventive factors such as physical activity and diet, and (4) treatment availability [11, 12]. The OKAT is a simple self-administered questionnaire that has been primarily validated to be used among English-speaking populations [12, 14].

While the OKAT was originally developed for healthy Australian women aged 25–44 years old, the questionnaire has been translated and used in several studies to ascertain the level of knowledge among people from Chinese-speaking backgrounds [15–17]. In a previous study, the OKAT was directly translated into Chinese to measure the knowledge levels of orthopaedic nurses in Hunan, China, and the tool could have been validated for its linguistic properties [17]. Considering the increased prevalence of migrants from Chinese-speaking countries residing in Australia, there is a need to culturally adapt the current OKAT for Australian–Chinese [8]. Given that OKAT has been developed in Australia, the tool makes it fit for the Australian–Chinese population and setting. The study aims to culturally adapt the OKAT for the Chinese-speaking diaspora in Australia according to Beaton and colleagues’ published guidelines for cross-cultural adaptation [18].

## Methods

**Fig. 1:**
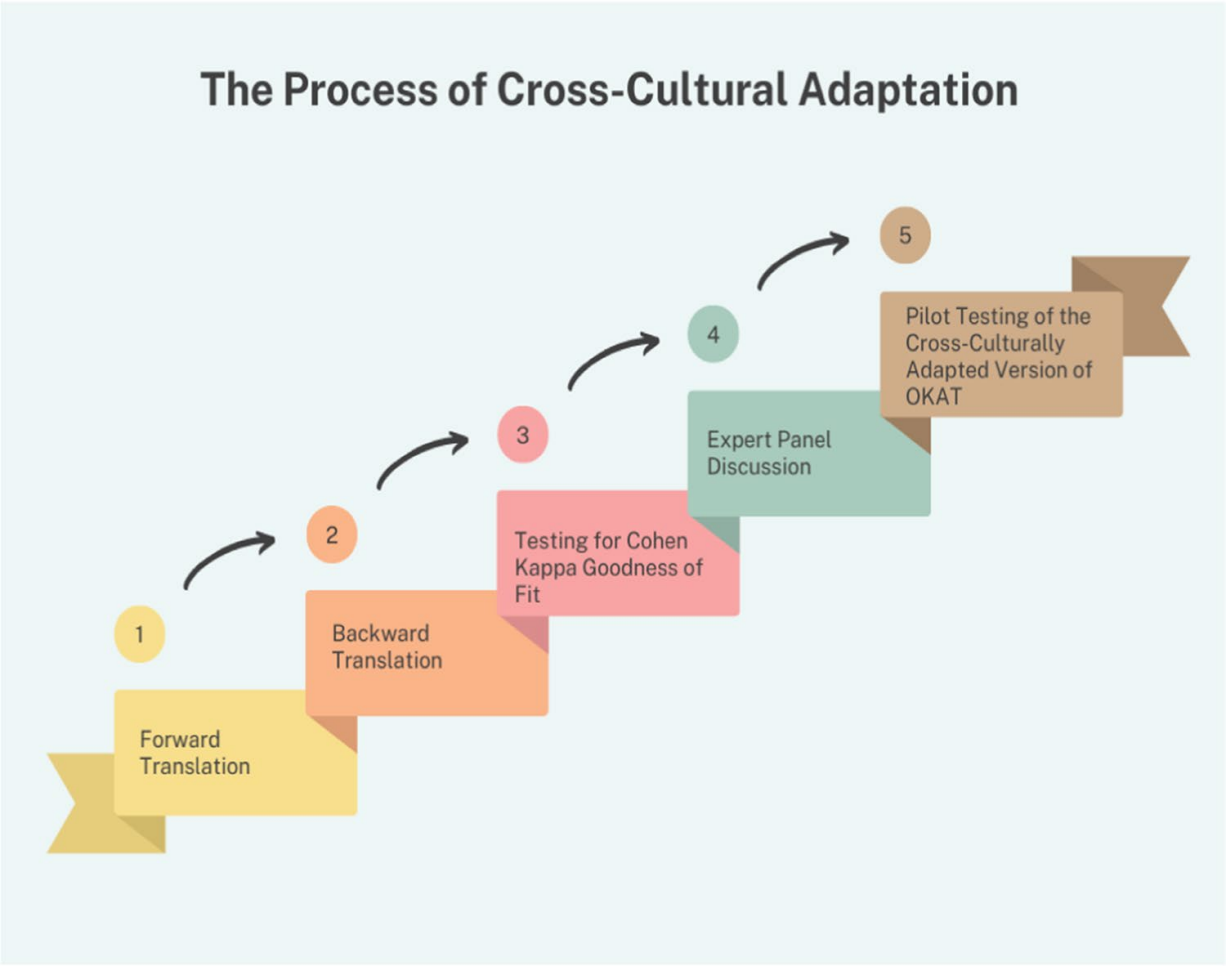
The process of cross-cultural adaptation according to the Beaton guidelines [18]

The study was conducted in five phases in 2023, in concordance with the published guidelines for cross-cultural adaptation [18] as outlined in Fig. 1. The study was approved by the Western Sydney University Ethics Committee (H13831).

### Phase 1: Forward translation

A professional interpreter certified by the National Accreditation for Translators and Interpreters conducted the forward translation of the tool. The professional translator is naïve to the original version of OKAT and is a registered allied health professional. Thus, the intent of phase 1 is to translate the English version of OKAT into simplified Chinese.

### Phase 2: Backward translation

Two individual language experts carried out the back translation, each with their own versions, T1 and T2, respectively. A meeting between two translators was conducted to achieve consensus and produce the synthesized back translation of the tool (T3).

### Phase 3: Testing for Cohen’s kappa goodness of fit

Participants aged 18 years and above who had at least high school proficiency in English and simplified Chinese were randomly recruited. Socio-demographic data of the participants were not captured in this stage of the research as the aim is to test the goodness of fit and the readability of the tool for the targeted ethnic group. Participants were asked to complete the two versions of the OKAT (original in English and translated version). Participants answered both versions of the tool in the order of their first language. The item agreement between the original and translated version of the OKAT was analyzed using weighted Cohen’s kappa on Stata (Version 18) licensed to the university. The interpretation of Cohen’s kappa results is defined according to values such as poor agreement if less than 0.20, fair agreement at a range between 0.20 and 0.40, moderate agreement at a range between 0.40 and 0.60, good agreement at a range between 0.60 and 0.80, and very good agreement at a range between 0.80 and 1.00 [19].

### Phase 4: Expert panel discussion

An expert committee consisting of two bilingual community workers, two language experts, and a postgraduate student discussed each item in the translated version of OKAT, with emphasis on items that were identified to require further discussion from the results obtained in the earlier phases. The goal of the expert committee was to obtain cross-cultural equivalence while maintaining the structure of the original OKAT when adapted to simplified Chinese. The committee discussed the translation according to its grammatical accuracy, readability, and understandability. A consensus was achieved regarding confusing or ambiguous items, and certain items were rephrased to reflect cultural sensitivity and language appropriateness to the target population. The cross-culturally adapted version was finalized for evaluation and pilot testing.

### Phase 5: Pilot testing of the cross-culturally adapted OKAT on three focus group discussions

The participants in the pilot testing were recruited using convenience sampling from a cohort of Chinese-speaking males and females who have participated in a community osteoporosis awareness program conducted in the Greater Western Sydney region. Participants in the study were given a copy of the simplified Chinese version of OKAT at the end of the program for completion. Apart from completing the questionnaire, participants were required to assess each item for its readability and understandability. For the purpose of the study, *readability* is defined as the quality of being legible and desirable. This was measured on a binary “Yes” or “No” scale. *Understandability* is defined in the study as the quality of being reasonably perceivable of its intended meaning and context. This was also measured on a binary “Yes” or “No” scale. Each item in the simplified Chinese version of OKAT was discussed with the participants, bilingual community health workers, and the researcher (CT). Another research member also observed the sessions (JB). The data obtained from the participants’ responses were analyzed using descriptive analysis.

## Results

### Phase 1 and phase 2

The forward translation of the OKAT to the simplified Chinese version was produced. In phase 2, a consensus was reached that the forward translation, when back-translated, reflects the semantic, idiomatic, experiential, and conceptual equivalence of the tool.

### Phase 3

A total of 33 participants were recruited in this phase. The participants were Mandarin-speaking and/or Cantonese-speaking, reflecting the latest census data that Mandarin is the most common non-English language spoken in Australia (2.7% of the population: *n* = 685,274) and that Cantonese is the most predominant Chinese dialect in the country (*n* = 295,281). The majority of the participants were female, aged 50 years and over, with education up to high school. The education level is congruent with the last 2019 Australian migrant survey.

The overall item agreement was 72%, with a weighted kappa of 0.55, indicating a moderate agreement between the original version of OKAT and the simplified Chinese version of OKAT (Table 1).

**Table 1:** Responses on the original version and simplified Chinese version

Original version	Simplified Chinese version *n* (%)			
	True	False	I don’t know	Total
True	250 (37)	36 (5)	44 (6)	330 (50)
False	33 (5)	127 (19)	28 (4)	188 (28)
I don’t know	22 (3)	23 (3)	96 (14)	141 (21)
Total	305 (46)	186 (28)	168 (25)	659 (100)

### Phase 4

No item was omitted in the cross-cultural adaptation process to retain the tool’s equivalence. A discussion was held about rephrasing the instructions in the tool. The panel highlighted that the word “questions” may be too confusing as the items are grammatically constructed as statements. The statements cannot be rephrased into questions (although this was suggested by one of the bilingual community workers) as this will lose the equivalence of the tool. A consensus was reached to rephrase the instruction, specifically simplifying the words used and focusing on how to rate the statements accordingly.

Out of 20 translated items, 12 items (2, 4, 5, 8, 9, 10, 12, 13, 14, 15, 16, and 17) were graded in consensus as accurately translated, readable, and understandable for the target population. The direct translation of seven items (3, 6, 7, 9, 11, 18, and 20) impacted the readability and understandability of the statements and therefore had to be culturally adapted. The summary of the item discussions is listed in Table 2 for context.

**Table 2:** Summary of items to be cross-culturally adapted

Item	Ambiguous words/phrases identified	Resolution
3. Having a higher peak bone mass at the end of childhood gives no protection against the development of osteoporosis in later life	“end of childhood” “peak bone mass”	The phrase “end of the childhood” was not clearly defined and could be interpreted in several ways considering the geographic background of the individual answering the tool (e.g., South China may interpret this either as “before or after period for girls”; “childhood” can also be perceived as young adolescent; gender specificity issues, e.g., “boys” and “girls” have different stages of childhood according to the culture and customs of an individual The phrase “peak bone mass” cannot be understood
6. White women are at highest risk of fracture as compared to other races	“White women”	The direct translation of “White” can raise ethical concerns against the race being described
7. A fall is just as important as low bone strength in causing fractures	“bone strength”	The term “bone strength” may not be understood
9. From age 50, most women can expect at least one fracture before they die	“die”	The word “die” is confronting, e.g., taboo, too depressing
11. It is easy to tell whether I am at risk of osteoporosis by my clinical risk factors	“clinical risk factors”	The direct translation of “clinical risk factors” was readable and grammatically correct, but too difficult to understand
18. There is a small amount of bone loss in the ten years following the onset of menopause	“menopause”	Males may not understand the direct translation of menopause
20. There are no effective treatments for osteoporosis available in Australia	The statement has to be rephrased	The item had to be rephrased for understandability purposes, expounding on treatment availability

### Phase 5

The readability and understandability ratings of the cross-culturally adapted OKAT by Chinese community members across the three workshops are summarized in Table 3. The line items left unanswered by the participants were accounted for as missing data. A total of 72 participants were recruited in this phase. The 33 participants were recruited to complete the translated version, while 39 participants were recruited to complete the cross-culturally adapted version.

**Table 3:** Readability and understandability rating of the cross-culturally adapted OKAT by participants across all workshops (*n* = 56)

Items in the OKAT	Readability *n* (%)			Understandability *n* (%)		
	Yes	No	Missing data	Yes	No	Missing data
1. Osteoporosis leads to an increased risk of fractures	50 (89)	0 (0)	6 (11)	46 (82)	0 (0)	10 (18)
2. Osteoporosis usually causes symptoms (e.g., pain) before fractures occur	36 (64)	14 (25)	6 (11)	41 (73)	4 (7)	11 (20)
3. Having a higher peak bone mass at the end of childhood gives no protection against the development of osteoporosis in later life	33 (59)	10 (18)	13 (23)	32 (57)	10 (18)	14 (25)
4. Osteoporosis is more common in men	29 (52)	20 (36)	7 (13)	36 (64)	8 (14)	12 (21)
5. Cigarette smoking can contribute to osteoporosis	37 (66)	13 (23)	6 (11)	43 (77)	3 (5)	10 (18)
6. White women are at highest risk of fracture as compared to other races	35 (63)	14 (25)	7 (13)	38 (68)	6 (11)	12 (21)
7. A fall is as important as low bone strength in causing fractures	45 (80)	3 (5)	8 (14)	41 (73)	2 (4)	13 (23)
8. By age 80, the majority of women have osteoporosis	42 (75)	5 (9)	8 (14)	41 (73)	1 (2)	14 (25)
9. From age 50, most women can expect at least one fracture before they die	42 (75)	10 (18)	4 (7)	40 (71)	4 (7)	12 (21)
10. Any type of physical activity is beneficial for osteoporosis	40 (71)	8 (14)	8 (14)	39 (70)	4 (7)	13 (23)
11. It is easy to tell whether I am at risk of osteoporosis by my clinical risk factors	32 (57)	12 (21)	12 (21)	35 (63)	8 (14)	13 (23)
12. Family history of osteoporosis strongly predisposes a person to osteoporosis	46 (82)	1 (2)	9 (16)	41 (73)	0 (0)	15 (27)
13. An adequate calcium intake can be achieved from two glasses of milk a day	42 (75)	7 (13)	7 (13)	43 (77)	1 (2)	12 (21)
14. Sardines and broccoli are good sources of calcium for people who cannot take dairy products	48 (86)	1 (2)	7 (13)	43 (77)	1 (2)	12 (21)
15. Calcium supplements alone can prevent bone loss	34 (61)	15 (27)	7 (13)	40 (71)	4 (7)	12 (21)
16. Alcohol in moderation has little effect on osteoporosis	42 (75)	7 (13)	7 (13)	44 (79)	0 (0)	12 (21)
17. A high salt intake is a risk factor for osteoporosis	48 (86)	2 (4)	6 (11)	44 (79)	0 (0)	12 (21)
18. There is a small amount of bone loss in the ten years following the onset of menopause	38 (68)	10 (18)	8 (14)	41 (73)	3 (5)	12 (21)
19. Hormone therapy prevents further bone loss at any age after menopause	42 (75)	5 (9)	9 (16)	42 (75)	1 (2)	13 (23)
20. There are no effective treatments for osteoporosis available in Australia	36 (64)	6 (11)	14 (25)	39 (70)	4 (7)	13 (23)

Among the 56 participants who agreed to rate the cross-culturally adapted version, all participants rated item 1 as readable and understandable. Out of the remaining items, 11 items were rated by the majority of the participants (between 71 and 89% of total participants) as readable, while 16 items were rated by the majority as understandable (between 70 and 82% of total participants). Furthermore, the rest of the items received varied ratings (52 to 63%). The three items that received lower reading and understanding ratings were items 3, 4, and 11 (52–59% of participants).

The comparison of scores obtained between the translated version and the cross-culturally adapted version of the OKAT is summarized in Table 4. The comparison between the translated version and cross-culturally adapted correct responses was expressed as percentages rather than counts (due to the differences in the total number of participants who answered each version) in the fourth column of Table 4.

**Table 4:** Comparison of correct responses obtained between the translated OKAT and the cross-culturally adapted OKAT

Items in OKAT	Cross-culturally adapted version *n* = 39 (%)	Translated version *n* = 33 (%)	Differences in responses between cross-culturally adapted and translated version of OKAT (%)
1	36 (92)	29 (90)	2
2	15 (38)	11 (34)	4
7	36 (92)	25 (78)	14
12	35 (90)	23 (72)	12
14	36 (92)	26 (81)	11
18	16 (41)	8 (25)	16
*Items observed with more than 20% improvement when cross-culturally adapted*			
3	29 (74)	4 (13)	25
5	37 (95)	24 (75)	20
6	17 (44)	7 (22)	22
8	35 (90)	21 (66)	24
9	24 (62)	7 (22)	40
10	20 (51)	7 (22)	29
16	34 (87)	14 (44)	43
17	34 (87)	18 (56)	31
19	28 (72)	10 (31)	41
*Items observed with lower ratings when cross-culturally adapted*			
4	23 (59)	21 (66)	-7
11	18 (46)	18 (56)	-10
13	24 (62)	26 (81)	-19
15	20 (51)	23 (72)	-21
20	8 (21)	14 (44)	-23

Out of the 20-item statements in the OKAT, 15 items were observed to have improvement, nine of which were observed to have an improvement of greater than 20% in the participants’ correct responses between the cross-culturally adapted version and the translated version. However, items 4, 11, 13, 15 and 20 were observed to have a reduction in correct responses when comparing the cross-culturally adapted version versus the translated version (7 to 23%).

## Discussion

The cross-culturally adapted version of the OKAT has good face and content validity, as evaluated by the Chinese-speaking populations in Australia, and has a moderate item agreement compared to the original OKAT version. The study provided a process example of how to appropriately adapt a clinical instrument for people from Chinese-speaking backgrounds in Australia. The researchers demonstrated in this study that a simple direct (forward–backwards) translation of a clinical instrument may be insufficient to accurately reflect and adequately measure the levels of apprehension by the culturally and linguistically diverse patient community; ambiguities in the direct translation of a tool can potentially lead to a systematic bias in the results. A simple or direct translation may consider the elements of linguistic properties such as grammar but often fails to account for the necessity of cultural appropriateness in the process.

Through the study process, several items in the validated OKAT required rephrasing and further contextualisation for the ethnic end-users, as the original statements have a bias towards the monolithic Western understanding of health and the privy of individualism; simple translation alone has been shown to confound the understanding of the participants. For example, in item 9 of the OKAT (*From age 50 years, most women can expect at least one fracture before they die.*), the word “die” can be too confronting for people from Chinese-speaking backgrounds and considerably taboo for middle-aged individuals. Furthermore, while a double-negative in English has a positive connotation, its usage in Chinese may not be as well understood since the usage of a double-negative has the potential to generate either an affirmative or negative concord. The study finding is congruent and reflects similar findings from other studies translating OKAT to Arabic, Hungarian, and Serbian [11, 20–22], and it may help explain the observed differences in OKAT scores from the respective translated versions. A more nuanced cross-cultural adaption, such as the process employed in this study, is therefore required to meaningfully translate and adapt clinical instruments for use.

While there were some key items that have resulted in a significantly higher score in the understandability and readability of the item, some of the items on the questionnaire could not be further adapted to improve understandability as the scope of these questions required participants to have knowledge about the local health systems and gendered-specific differences. For example, item 20 (*There are no effective treatments for osteoporosis available in Australia*) requires the participants to have prior knowledge about the Australian Healthcare system, while item 18 (*There is a small amount of bone loss in the ten years following the onset of menopause*) requires male participants to understand what “menopause” is. Considering that osteoporosis impacts one in five males aged over 50 years old [23], it may be worth considering the gendered knowledge differences [23]. Furthermore, there is a need to consider the translatability of questions for its language, cultural and gender-diverse populations in future research when first developing new questionnaires to ascertain outcomes for people with osteoporosis.

With the Australian government’s increased focus on designing and delivering culturally inclusive health services and preventative health, future applications of our culturally adapted OKAT may include the incorporation into a health promotion toolkit to increase consumer awareness about osteoporosis.

### Strengths and limitations

This study demonstrated a rigorous cross-cultural adaptation of the validated OKAT for Australian–Chinese. The study has undertaken a rigorous process outlined by Beaton and colleagues’ published guidelines for cross-cultural adaptation [18]. This study also has some limitations. The small sample size and the inclusion of participants from the predominant Mandarin- and Cantonese-speaking backgrounds may limit the applicability of the findings to other Asian migrant groups in Australia. We did not capture the participants’ socio-demographic data, but these may have the potential to provide more nuanced insights into the impact on the understandability of the instrument. Nonetheless, this is the first study that has culturally adapted the OKAT for the Chinese-speaking diaspora in Australia.

## Conclusion

The cross-cultural adaptation of the OKAT to the simplified Chinese version has good face validity and is acceptable for use in the older Chinese-speaking populations in Australia.

## Data Availability

The data that support the findings of this study are available from the corresponding author, DL, upon reasonable request.
